# Burden and trends of symptomatic sexually transmitted infections in Malawi from 2000 to 2021: comparative analysis of survey and case report data

**DOI:** 10.1097/OLQ.0000000000001919

**Published:** 2024-01-03

**Authors:** Julia Michalow, Andreas Jahn, Anne Cori, Marie-Claude Boily, Tiwonge Chimpandule, Stone Mbiriyawanda, Washington Ozituosauka, Rose Nyirenda, Jeffrey W Imai-Eaton

**Affiliations:** 1 MRC Centre for Global Infectious Disease Analysis, School of Public Health, Imperial College London, London, United Kingdom; 2 Malawi Department of HIV, STI & Viral Hepatitis, Ministry of Health, Lilongwe, Malawi; 3 I-TECH Malawi, Lilongwe, Malawi; 4 Department of Global Health, University of Washington, Seattle, WA, USA; 5 Center for Communicable Disease Dynamics, Department of Epidemiology, Harvard T.H. Chan School of Public Health, Boston, MA, USA

**Keywords:** Sexually transmitted infection, syndromic management, surveillance, sub-Saharan Africa

## Abstract

**Background:**

In settings without aetiologic testing for sexually transmitted infections (STIs), programmes rely on STI symptom data to inform priorities. To evaluate whether self-reported STI symptoms in household surveys consistently represent the STI burden, we compared symptomatic infection rates between survey self-reporting and health facility case reporting in Malawi.

**Methods:**

We analysed self-reported symptoms and treatment seeking in the past year among sexually active adults from four Malawi Demographic and Health Surveys between 2000–2015. Bayesian mixed-effects models were used to estimate temporal trends, spatial variation, and sociodemographic determinants. Survey reporting was compared with health facility syndromic diagnoses between 2014–2021.

**Results:**

In surveys, 11.0% (95% CI:10.7–11.4%) of adults reported STI or STI-related symptoms in the last year, of whom 54.2% (52.8–55.7%) sought treatment. In facilities, the mean annual symptomatic case diagnosis rate was 3.3%. Survey-reported treatment in the last year was 3.8% (95% CrI:2.3–6.1%) for genital ulcer, 3.8% (2.0–6.7%) for vaginal discharge, and 2.6% (1.2–4.7%) for urethral discharge. Mean annual diagnosis rates at facilities were 0.5% for genital ulcer, 2.2% for vaginal discharge, and 2.0% for urethral discharge. Both data sources indicated a higher burden of symptoms among women, individuals above 25 years, and in Southern Malawi.

**Conclusion:**

Survey and facility case reports indicated similar spatial and demographic patterns of STI symptom burden and care seeking, but implied large differences in the magnitude and relative burden of symptoms, particularly genital ulcer, which could affect programme priorities. Targeted aetiologic surveillance would improve interpretation of these data to enable more comprehensive STI surveillance.

## Summary

Two data sources for surveillance of sexually transmitted infection symptoms in Malawi had similar spatial and demographic patterns but differed in the magnitude, trends, and relative burden of symptoms.

## Introduction

Sexually transmitted infections (STIs) are a substantial public health challenge worldwide, particularly in sub-Saharan Africa (SSA).^1^ Treating STIs and controlling transmission requires surveillance to monitor trends, identify higher burden populations and areas, and optimise programme implementation. However, STI surveillance is challenging in lower-resource settings.^2^ Aetiologic prevalence surveys are the preferred data source for STI burden assessment, but are expensive, infrequent, and often have limited geographic coverage.^2^ Different methodologies within and between countries also impede comparing results.^2^

In SSA, the most available data are on experience of STI symptoms through health facility syndromic case reports or household surveys recording self-reported symptom history. These sources, which are potentially more consistently available and population representative than sporadic aetiologic prevalence surveys, have not been used to monitor STI trends due to several limitations. Syndromic management (presumptive STI treatment based on clinical symptoms without laboratory confirmation) remains the norm in most settings due to limited routine laboratory testing capacity.^1^ Lack of case definition standardisation, incomplete and aggregated facility reporting (without age or gender stratification) have hindered the use of syndromic case data in STI burden assessment.^2^

Nationally-representative household surveys, such as quinquennial Demographic and Health Surveys (DHS),^3^ often measure self-reported STI symptom prevalence and treatment seeking, but without aetiologic STI testing. However, like facility case reporting, STI symptoms are often nonspecific and may reflect several infectious or non-infectious causes, such as bacterial vaginosis, and also omit high proportions of asymptomatic infection.^4^

This study explored using self-reported data on symptomatic infections to monitor STI burden and trends. We assessed the consistency of STI determinants, trends, and spatial patterns from self-reported symptoms in household surveys and facility-reported syndromic cases in health management information systems in Malawi and compared the distribution of symptoms across both datasets.

## Methods

### Data sources and study measures

We compared two data sources on symptomatic STIs in Malawi. The first was the Malawi DHS, conducted in 2000, 2004, 2010 and 2015–16.^3^ DHS were nationally representative cross-sectional household-based surveys. Women aged 15–49 years and men 15–54 years were eligible to participate who were permanent residents or visitors in selected households. All men in one-third of the sampled households were selected for the male survey. Variable definitions were informed by the Guide to DHS Statistics (Table S1).^5^

Outcomes analysed were self-reported genital ulcer, genital discharge, and known STI (defined in the DHS as infection acquired through sexual contact, other than HIV) in the last 12 months, and, among those, self-reported care or treatment sought for an STI or STI-related symptom in the last 12 months. Among those reporting care or treatment, the sector (public, private, or other) was also assessed for 2010 and 2015–16. Covariates, selected based on factors associated with STI infection in previous literature, included socio-demographic and behavioural characteristics, and STI-related knowledge.^6,7^ Socio-demographic variables were age (15–19, 20–24, 25–29, 30–24, 35–39, 40+ years), sex (male, female), place of residence (urban, rural), marital status (never married, formerly married, married or living together), education (none, primary, secondary or higher), and current employment. Behavioural variables were age at first sex (<15, 15–19, 20–24, 25+ years), number of sex partners in the last 12 months (none, one, two or more), condom use at last sex in the last 12 months (yes, no or unknown), and ever tested for HIV (yes, no or unknown). Knowledge of STIs was measured as knowing diseases can be transmitted through sexual contact (yes, no or unknown).

The second data source was health facility-level aggregate STI syndrome case counts recorded in the Malawi Department of HIV and AIDS Management Information System (DHAMIS), a database recording public sector supported HIV and related services in Malawi, provided through clinics, health posts, dispensaries, health centres, and hospitals, among others. Each quarter, monthly STI case reports are manually aggregated from paper STI clinic registers and verified.^8^ We analysed data between January 2014 and December 2021. During this period, reporting facilities increased gradually from 708 in 2014 to 794 in 2021. Aggregate STI case reports were stratified by sex (male, female), age (0–19, 20–24, 25+ years), and HIV status (HIV-positive, HIV-negative, unknown). STI diagnoses, which were not age-stratified, included genital ulcer disease (GUD), urethral discharge syndrome (UDS), low-risk vaginal discharge syndrome (VDS), high-risk VDS, lower abdominal pain, among others. As diagnoses could include multiple syndromes, the number of diagnoses was typically larger than the number of clients (Figure S1). Outcome variables for this analysis were cumulative annual diagnoses of GUD, UDS, and VDS (low- and high-risk combined) to align with DHS outcomes.

Adjusted district-level population estimates by age-group, sex, and year for 2014–2021 were used as denominators for STI diagnosis rates. The Malawi National Statistics Office projected population totals by district, sex, and five-year age-group from the 2008 household census, accounting for anticipated population growth. These annual projections were adjusted proportionally in each district, sex, and age-group to align with national census results in 2018.^9,10^

### Analysis

DHS respondent characteristics and self-reported STI and treatment seeking were summarised as frequencies and survey-weighted proportions with design-based standard errors and 95% confidence intervals (95% CI). Treatment seeking by health sector was assessed using only the 2010 and 2015–16 DHS, due to inconsistent questionnaire formulation in earlier surveys.

To identify factors associated with self-reported STI symptoms, we used four Bayesian logistic mixed-effects models to estimate the odds of self-reported genital ulcer and genital discharge in the last 12 months among ever sexually active males and females. To identify factors associated with STI treatment seeking, two Bayesian logistic mixed-effects models were used to estimate the odds of treatment seeking among males and females reporting an STI or symptoms in the last 12 months. All models included random intercepts and slopes over calendar year for the 33 geographic areas (29 health districts and four metropolitan cities). Survey cluster-level random effects were included to account for the clustered sampling design.^5^ Fixed effects included covariates for sociodemographic and behavioural characteristics, and STI-related knowledge. The models did not use sampling weights.

Health facility syndromic case reports in DHAMIS were checked for consistency and completeness. Severely high outliers, identified as monthly cases five times larger than the third quartile for the relevant health facility, year, and diagnosis, were assumed to be reporting errors and replaced with the median number of diagnoses per month for the corresponding health facility and year. The annual rate of syndromic diagnoses was calculated per district and year by dividing total number of diagnoses of each syndrome across all ages by the adult population aged 15–54 years.

To assess similarities in the distribution and characteristics of syndromic diagnoses in health facility case reports and STI care seeking in the past year in household surveys, we estimated the probability of treatment for genital ulcer and genital discharge in the last 12 months among all adult DHS respondents. Bayesian logistic mixed-effects models included random intercepts and slopes over calendar year for each geographic area and fixed effects for year and age interacted with sex. Models were used to predict the proportion of all adults aged 15–54 years seeking treatment for each symptom in the last year at district, region, and national level for the period 2014–2021, assuming a flat trend after the year 2015 (the year of the final survey). To compare the extent of district-level variability between data sources, we calculated coefficients of variation for each symptom based on average district rates during 2014–2021.

Results were presented as adjusted odds ratios (aORs) with 95% credible intervals (95% CrI). All regression models used diffuse priors. Analyses were performed in R version 4.2.1, using the rstanarm package.^11^

Ethical approval for this study was granted by the Imperial College Research Ethics Committee (ICREC #6365329).

## Results

### Self-reported STI symptoms and treatment seeking in household surveys

**Figure 1:**
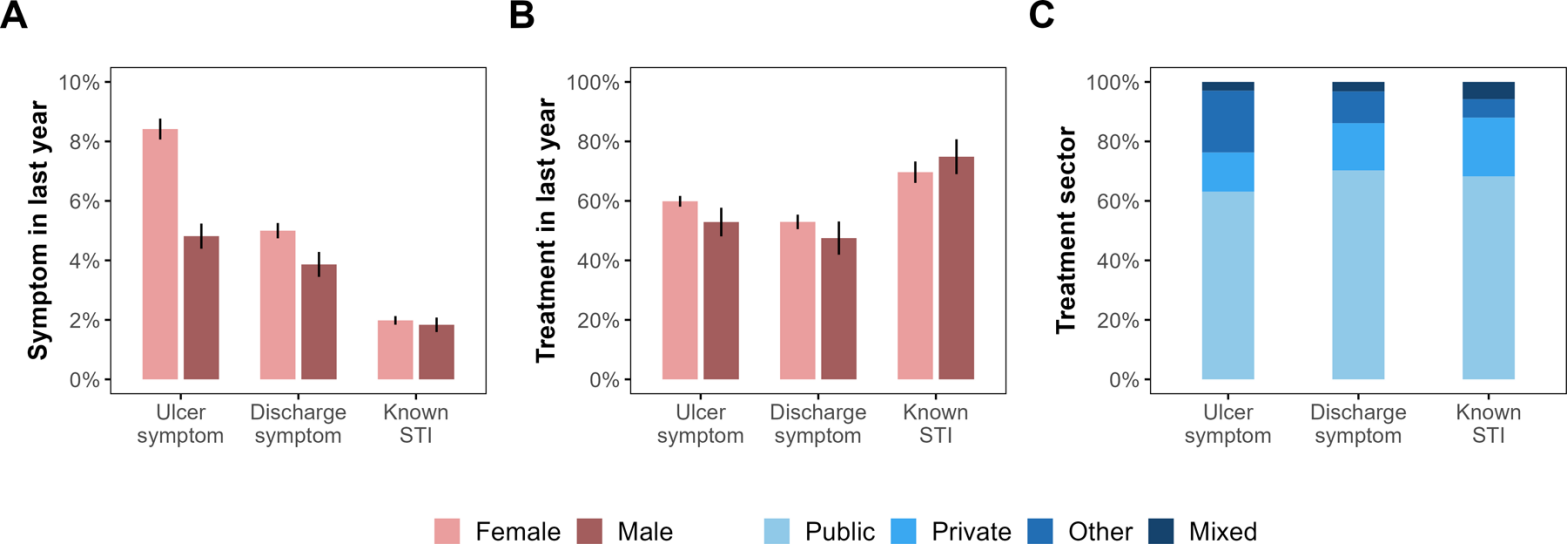
Percentage of ever sexually active adult DHS respondents self-reporting (A) STI or STI-related symptoms in the last year during 2000, 2004, 2010 and 2015–16, (B) treatment seeking for an STI or STI-related symptom in the last year during 2000, 2004, 2010 and 2015–16 among adults who reported STI or STI-related symptoms, and (C) health sectors accessed for STI treatment in the last year in 2010 and 2015–16. Vertical line segments in (A) and (B) represent 95% confidence intervals.

The four DHS included 60,039 women aged 15–49 years and 17,703 men aged 15–54 years who reported having ever had sex (Table S2). During 2010 and 2015–16, 11.0% (95% CI: 10.7–11.4%) of adults reported having an STI or STI-related symptom in the last year, of whom 54.2% (52.8–55.7%) sought treatment. More women than men reported genital ulcer (8.4% [8.1–8.8%] vs. 4.8% [4.4–5.2%]) and genital discharge (5.0% [4.7–5.3%] vs. 3.9% [3.5–4.3%]; Figure 1A). Among those reporting symptoms, women were slightly more likely to seek treatment than men (55.3% [53.8–56.9%] vs. 48.8% [44.9–52.7%]; Figure 1B).

Among those seeking treatment, 64.8% (62.3–67.3%) used the public sector, 15.0% (13.2–16.8%) the private sector, and 17.3% (15.4–19.3%) other services (Figure 1C). This distribution was similar across symptoms, although a higher proportion of adults with ulcer reported seeking other treatment means. More men than women (28.1% [22.4–33.7%] vs. 12.8% [11.0–14.6%]) sought treatment in the private sector.

Factors associated with reporting an STI-related symptom varied by sex (Table 1). Among women, reporting ulcer and discharge were higher among those formerly married than never married (aOR: 1.45 [1.23–1.73]; aOR: 1.33 [1.09–1.62]), primary school educated than never educated (aOR:1.27 [1.16–1.40]; aOR: 1.25 [1.10–1.41]) and employed than unemployed (aOR:1.13 [1.06–1.21]; aOR: 1.18 [1.08–1.29]). Women aged 35–39 years were most likely to report genital ulcer (aOR: 1.15 [1.03–1.28]), compared to 25–29 years. Among men, only primary school education (aOR: 1.50 [1.09–2.12]) was associated with ulcer and employment (aOR: 1.38 [1.09–1.76]) was associated with discharge. Men (aOR: 2.26 [1.63–3.15]; aOR: 2.36 [1.66–3.33]) and women (aOR: 2.73 [2.14–3.49]; aOR: 2.93 [2.16–3.98]) with two or more sex partners in the last year were much more likely to report ulcer and discharge than those with no partners. As expected, the odds of reporting either symptom was higher among men and women who reported having an STI in the last year.

Spatially, STI symptom reporting was higher in Central and Southern Malawi (Figure S2). The odds of ulcer were almost four times higher among women in the Central (aOR: 3.62 [2.67–4.82]) and Southern (aOR: 3.63 [2.71–4.80]) than Northern region, and about two times higher among men (Central aOR: 1.98 [1.25–3.13]; Southern aOR: 2.36 [1.52–3.71]). There was no evidence of linear time trend in symptom reporting among women (ulcer aOR: 1.00 [0.98–1.02]; discharge aOR: 1.01 [0.99–1.03]) or men (ulcer aOR: 1.02 [0.99–1.04]; discharge aOR: 1.01 [0.98–1.03]), and there was no consistent temporal trend across and within sub-regional areas (Figure S3).

Treatment seeking, among those reporting an STI or STI-related symptom, was higher for urban than rural residents (women aOR: 1.43 [1.21–1.69]; men aOR: 1.69 [1.19–2.43]), and women with ulcers rather than discharge (aOR: 1.70 [1.50–1.94]) (Table S3). The odds of treatment decreased by 5% per year (aOR: 0.95 [0.93–0.97]) for women and 4% (aOR: 0.96 [0.92–1.00]) for men but did not vary substantially by region or district (Figures S4 and S5).

### Facility-reported syndromic case diagnoses

Between 2014 and 2021, 2,547,737 STI client case reports were recorded in DHAMIS (Table S4). Most cases (96.7%) were reported by facilities in the public sector. 59.4% were female, 67.7% were over 25 years of age, and 11.2% were asymptomatic partners. The majority (74.6%) of clients had not been treated previously for an STI. The Southern and Central regions recorded 51.9% and 34.8% of total cases, respectively, compared to 43.3% and 43.8% of the adult population in 2021. Of 2,657,076 syndromic diagnoses, the highest proportions were for VDS (30.2%), UDS (25.4%), GUD (14.0%), and lower abdominal pain (13.9%).

**Figure 2:**
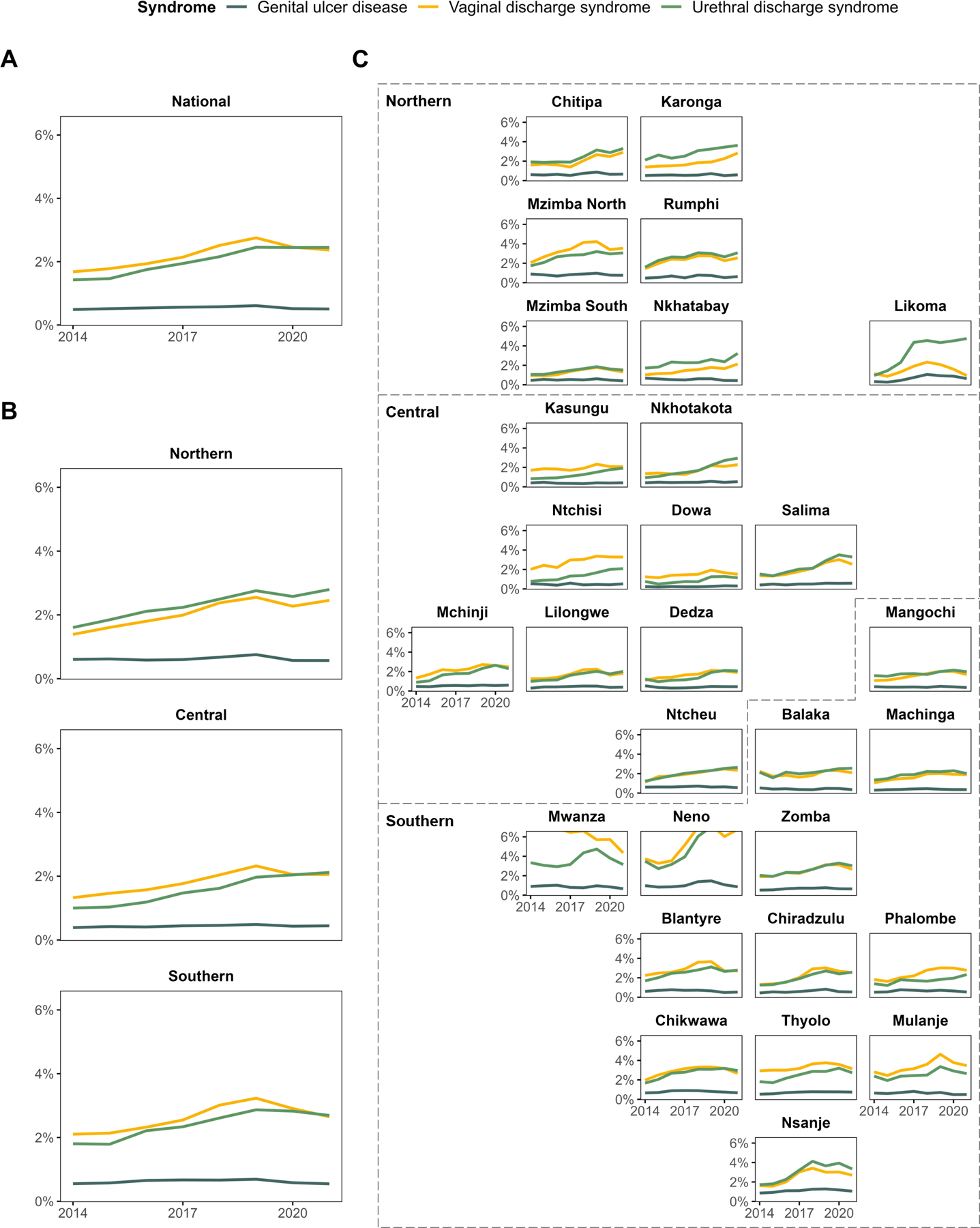
Annual rate of syndromic case diagnoses among adults aged 15–54 years during 2014–2021 at (A) national, (B) regional, and (C) district level according to geographic proximity. Diagnosis rates were calculated using total reported syndromic diagnoses and sex-matched census population estimates per year per district. *Y-axes truncated at 6%; maximum rate of 9.7% (2014) for vaginal discharge syndrome in Mwanza, and 7.2% (2019) and 7.3% (2021) for vaginal discharge and urethral discharge syndromes in Neno.

Nationally, the annual case diagnosis rate among adults 15–54 years between 2014–2021 was 3.7% for all cases and 3.3% for symptomatic cases. The rate of VDS diagnoses among adult women increased from 1.7% in 2014 to 2.4% in 2021. The rate of UDS diagnoses increased from 1.4% to 2.5%. GUD annual diagnosis rates consistently averaged 0.5% over time (Figure 2A). Southern and Northern Malawi had the highest case rates across all three diagnoses (Figure 2B). The mean case diagnosis rates were particularly high for VDS and UDS in Mwanza (6.7% and 3.6%) and Neno (5.4% and 5.2%) in the South and for UDS in Likoma (3.5%), a small island district in Lake Malawi (Figure 2C).

### Comparison of facility-reported syndromic case diagnoses and survey-reported treatment seeking

In household surveys, most adults reporting treatment for genital ulcer and genital discharge in the last year were female (67.5% [66.3–67.5%]; 62.0% [60.8–63.6%]) and over 25 years (73.1% [72.7–73.4%]; 68.2% [68.0–68.4%]). These were broadly consistent with the sex distribution (59% female) and age distribution (68% over 25 years) recorded in health facility case reporting.

**Figure 3:**
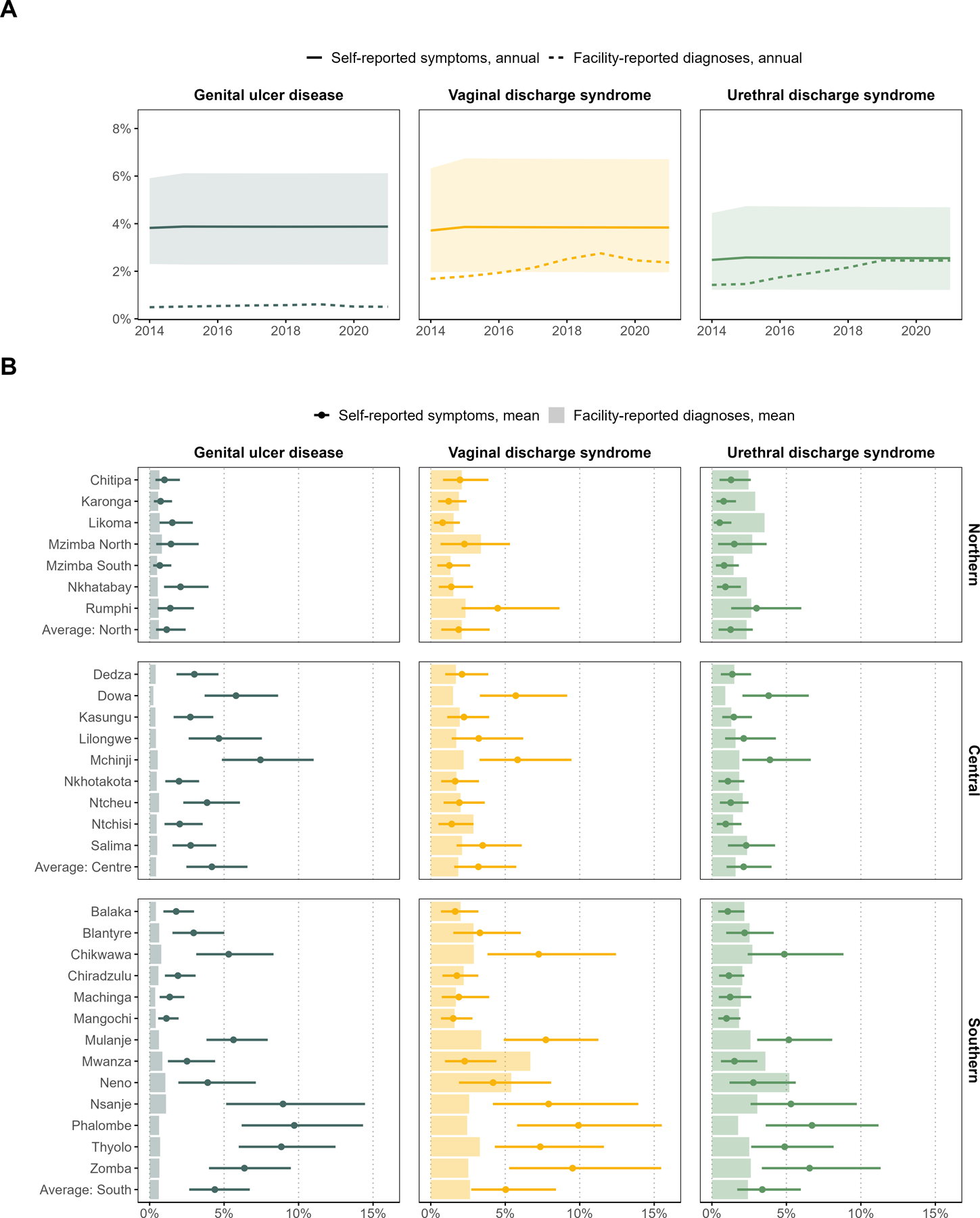
Comparison of the prevalence of individuals with self-reported symptoms who sought treatment in the last 12 months and annual facility-reported syndromic diagnosis rates, among adults aged 15–54 years in Malawi during 2014–2021. (A) National level comparison of annual rates per symptom. Dotted lines represent aggregate health facility case reports. Solid lines represent self-reported symptoms from household surveys between 2000 and 2015–16, predicted to 2014–2021. Shading represents 95% credible intervals around self-reported symptoms. (B) District and regional level comparison of mean rates per symptom. Thick solid bars represent aggregate health facility case reports. Points represent self-reported symptoms from household surveys between 2000 and 2015–16, predicted to 2014–2021. Error bars represent 95% credible intervals around self-reported symptoms.

In household surveys, nationally, 3.8% (95% CrI: 2.3–6.1%) of adults reported care seeking for genital ulcer in the last year, 3.8% (2.0–6.7%) for vaginal discharge, and 2.6% (1.2–4.7%) for urethral discharge. Although lower, the annual rate of health facility syndromic diagnoses among adults was within the credible intervals of the survey predictions for urethral discharge and vaginal discharge (Figure 3A). However, the average facility diagnosis rate for genital ulcer was much lower (0.5%) than treatment seeking proportions reported in the surveys.

Discrepancies between self-reported and facility-reported measures averaged during 2014–2021 were most striking in Central and Southern Malawi (Figure 3B). In these regions, self-reported treatment for genital ulcer was 9.6 times higher (4.2% [2.5–6.6%] vs. 0.4%) and 7.1 times higher (4.4% [2.7–6.7%] vs 0.6%) than facility-reported diagnosis rates. Vaginal discharge self-reporting was 1.7 (3.2% [1.6–5.7%] vs. 1.9%) and 1.9 (5.0% [2.7–8.4%] vs. 2.6%) times higher and urethral discharge self-reporting was 1.3 (2.1% [1.0–4.0%] vs. 1.6%) and 1.4 (3.4% [1.7–6.0%] vs. 2.4%) times higher than facility case diagnosis rates in the Central and Southern regions.

Coefficients of variation, averaged across all symptoms, indicated higher district-level variation for survey self-reported treatment (74.4%) than facility case diagnosis rates (38.6%). District-level variation was highest in the South, in both survey self-reporting (South: 64.5% vs. Central: 53.1% and Northern: 56.8%) and facility case-reporting (38.5% vs. 23.5% and 25.3%).

## Discussion

Nationally representative household surveys and health facility case reporting in Malawi indicated divergent rates, distributions, and temporal trends in STI symptoms. In household surveys between 2000 and 2015–16, around 11% reported having an STI or STI-related symptom in the last year, of whom around half sought treatment for their last infection, implying that 6.0% of adults per year present for STI care. In health facility case reports between 2014 and 2021, the mean annual symptomatic case diagnosis rate was only 3.3%.

Symptom distribution also differed between data sources. In surveys, genital ulcer and vaginal discharge were most common among adults seeking treatment. In national case reports, facility diagnosis rates were highest for vaginal discharge and urethral discharge syndromes, and substantially lower for genital ulcer. This diagnostic distribution aligns with previous research among STI clients attending an urban and rural hospital in Malawi.^12,13^ Furthermore, surveys indicated a higher proportion of symptomatic women treated for genital ulcer than men. The absence of sex-stratified case report diagnoses precluded direct comparison, but prior research identified the opposite sex trend among STI clients.^12,13^ It is unlikely that the 90% lower GUD rate recorded in health facility data from 2014–2021, compared to survey data from 2000–2015, is explained by the decreasing prevalence of ulcerative STIs (HSV-2, syphilis, and chancroid) over time in SSA.^14–16^

STI care seeking outside predominantly public sector facilities reporting to the national health information system may partly account for differences in the magnitude and distribution of symptoms across data sources. In 2021, DHAMIS case-reporting included 794 health facilities. The 2018 master facility list documented 963 public, private, and traditional STI service providers in Malawi, but this likely underestimated the count due to difficulties capturing unregistered or informal sources of care.^17^ Most survey participants reported seeking care in the public health sector, but over 30% sought treatment from the private and traditional sectors. Men reported using the private sector more than women. This was consistent with previous research that men in SSA prefer private facilities for perceived increased quality of care and privacy, despite higher costs.^18–20^ Individuals with genital ulcer reported using other means of treatment, such as traditional healers, more often than those with other symptoms. Although the traditional health sector is widely used in Malawi,^21^ differences in traditional care-seeking by STI symptom have not been previously documented. Moreover, frequent antibiotic stockouts in the public sector may result in periods of increased reliance on private and traditional providers.^22^ We were however unable to evaluate these patterns.

Temporal trends differed between the two data sources, though our assessment was limited by not having survey data after 2015 for direct comparison with health facility data. While facility case diagnosis rates increased between 2015 and 2020, household surveys showed no significant change in STI symptom reporting and a decline in STI care seeking between 2000 and 2015. The trends could have reversed, particularly trends in care seeking coinciding with health system improvements supported through HIV service decentralisation. One possible explanation for the rise in syndromic diagnoses is the large scale-up of HIV testing in Malawi since 2015,^23^ which may have facilitated STI detection and management, underscoring the importance of integrated health service provision.^1^ The upcoming Malawi DHS will help assess if increasing facility diagnoses are reflected in self-reporting, and if so whether they are attributable to higher rates of symptomatic infection or treatment seeking.

Despite discrepancies in overall levels, both data sources had similar demographic and spatial patterns, consistent with the burden of STIs. They indicated a higher burden of symptoms among women, individuals above 25 years, and in Southern Malawi. Specifically, self-reported genital ulcer in surveys was highest among women 35–39 years old and in the Southern region, aligning with the prevalence of HSV-2 and HIV. HSV-2, the main cause of genital ulcer in SSA,^14^ is more prevalent among women within this older age group due to the lifelong nature of infection.^24^ HIV prevalence in Malawi is also higher among women aged 35–54 years and in the South.^25^ These complementary trends suggest household surveys can provide insight into the relative prevalence of symptomatic STIs. However, future analyses aiming to reconcile the burden and distribution of STI symptoms with STIs should incorporate factors influencing the aetiology of these symptoms. For instance, a recent study among STI clinic attendees with genital ulcer in Malawi identified a substantial proportion of cases attributed to syphilis, indicative of shifts in genital ulcer aetiology over time, and a considerably higher proportion of HSV-attributed cases among those HIV-positive than HIV-negative.^26^

District level differences between survey self-reporting and facility case-reporting require further investigation to inform programme planning and prioritisation. Districts with high self-reported treatment but low facility diagnoses might rely more on the private and traditional sectors for STI service provision. Programmes should examine this further and consider expanding public sector services in these areas to address potential unmet treatment needs. Furthermore, district-level variation was greater for self-reported treatment than facility case diagnoses. Surveys may thus better identify districts with a higher infectious burden for prioritised programming.

Limitations in the two data sources may contribute to the identified inconsistencies. Self-reporting in household surveys may under-represent symptomatic infection, due to recall and social desirability biases,^27^ and potential lack of awareness of STI symptoms.^28,29^ Over-reporting may have resulted from using nonspecific questions on STI symptoms. Our analysis of routine case reporting may have overestimated syndromic diagnosis rates among adults, due to limited stratification available in DHAMIS. We included diagnoses across all age groups, yet 8.8% of total diagnoses were among those younger than 19 years. Additionally, clients may have been double counted if they sought care from multiple health facilities or experienced recurrent symptoms in a year.

In conclusion, in Malawi, where aetiologic STI surveillance is not widely implemented, we identified non-negligible discrepancies in the magnitude, trends, and distribution of STI symptoms between household survey self-reporting and health facility syndromic case reporting. Spatio-demographic trends were however similar across data sources, suggesting that survey self-reporting could aid programme prioritisation and investment in the absence of aetiologic prevalence data and reliable case-reporting data. The analysis did not account for asymptomatic infections, which constitute a high proportion of the overall infectious burden. Localised STI sentinel surveys could help adjudicate some of the inconsistencies identified, such as the different relative burden of STI symptoms, to facilitate interpretation of both data sources for a more comprehensive understanding of STI epidemiology and surveillance.

## Supplementary Material

Supplementary Material

## Figures and Tables

**Table 1:** Bayesian generalised linear mixed-effects models on the odds of reporting genital ulcer and genital discharge in the last 12 months, among ever sexually active males and females aged 15–54 years.

	Ulcer		Discharge	

	Female aOR (95% CrI)	Male aOR (95% CrI)	Female aOR (95% CrI)	Male aOR (95% CrI)
**Intercept**	0.01 (0.01–0.02)	0.01 (0.00–0.02)	0.01 (0.01–0.02)	0.02 (0.01–0.04)
**Year** (centred at 2010)	1.00 (0.98–1.02)	1.02 (0.99–1.04)	1.01 (0.99–1.03)	1.01 (0.98–1.03)
**Age group**				
15–19	0.69 (0.60–0.79)	1.11 (0.80–1.55)	1.08 (0.93–1.26)	1.25 (0.86–1.84)
20–24	0.82 (0.74–0.90)	0.95 (0.72–1.23)	0.96 (0.87–1.08)	1.08 (0.80–1.48)
25–29	Ref	Ref	Ref	Ref
30–34	1.12 (1.01–1.23)	1.06 (0.82–1.35)	1.06 (0.93–1.20)	0.94 (0.67–1.31)
35–39	1.14 (1.03–1.27)	1.05 (0.79–1.39)	1.08 (0.94–1.24)	1.08 (0.76–1.51)
40+	0.88 (0.79–0.98)	0.93 (0.73–1.21)	0.9 (0.78–1.03)	0.66 (0.47–0.91)
**Residence type**				
Rural	Ref	Ref	Ref	Ref
Urban	1.14 (1.02–1.27)	1.24 (0.98–1.54)	1.06 (0.93–1.22)	1.07 (0.80–1.40)
**Region**				
Northern	Ref	Ref	Ref	Ref
Central	3.62 (2.67–4.82)	1.98 (1.25–3.13)	1.68 (1.28–2.20)	1.71 (1.06–2.80)
Southern	3.63 (2.71–4.80)	2.36 (1.52–3.71)	1.71 (1.31–2.24)	1.41 (0.88–2.28)
**Marital status**				
Never married	Ref	Ref	Ref	Ref
Formerly married	1.45 (1.23–1.73)	1.17 (0.79–1.70)	1.33 (1.09–1.62)	0.63 (0.38–1.03)
Married or living together	1.28 (1.08–1.51)	0.83 (0.62–1.13)	1.15 (0.96–1.39)	0.43 (0.31–0.60)
**Highest education**				
None	Ref	Ref	Ref	Ref
Primary	1.27 (1.16–1.40)	1.5 (1.09–2.12)	1.25 (1.10–1.41)	1.18 (0.82–1.72)
Secondary or higher	1.09 (0.96–1.25)	0.99 (0.69–1.44)	0.94 (0.79–1.11)	0.74 (0.50–1.13)
**Employment**				
No or unknown	Ref	Ref	Ref	Ref
Yes	1.13 (1.06–1.21)	1.09 (0.88–1.36)	1.18 (1.08–1.29)	1.38 (1.09–1.76)
**Age at first sex**				
<15	1.04 (0.96–1.13)	1.15 (0.96–1.39)	1.05 (0.95–1.17)	1.21 (0.97–1.51)
15–19	Ref	Ref	Ref	Ref
20–24	0.94 (0.83–1.05)	0.83 (0.67–1.02)	0.93 (0.80–1.08)	0.83 (0.63–1.09)
25+	0.75 (0.50–1.09)	0.69 (0.43–1.08)	0.94 (0.57–1.46)	1 (0.56–1.68)
**Number of sex partners in past 12 months** ^1^				
None	Ref	Ref	Ref	Ref
1	1.2 (1.06–1.35)	1.15 (0.84–1.55)	1.48 (1.26–1.73)	1.29 (0.94–1.79)
2+	2.73 (2.14–3.49)	2.26 (1.63–3.15)	2.93 (2.16–3.98)	2.36 (1.66–3.33)
**Condom use at last sex in past 12 months** ^1^				
No or unknown	Ref	Ref	Ref	Ref
Yes	1.38 (1.23–1.56)	0.87 (0.69–1.10)	1.21 (1.06–1.40)	0.81 (0.63–1.04)
**Ever tested for HIV**				
No or unknown	Ref	Ref	Ref	Ref
Yes	1.15 (1.04–1.27)	1.19 (1.00–1.42)	1.11 (0.98–1.25)	1.04 (0.84–1.29)
**Knowledge of STIs**				
No or unknown	Ref	Ref	Ref	Ref
Yes	0.98 (0.87–1.10)	1.28 (0.90–1.85)	0.71 (0.62–0.81)	0.83 (0.58–1.20)
**Any STI in past 12 months** ^2^				
No or unknown	Ref	Ref	Ref	Ref
Yes	11.4 (10.0–13.0)	18.1 (14.2–23.1)	17.8 (15.3–20.5)	36.3 (27.2–48.4)
**Random effects**				
τ_00_ geographic area	0.28	0.37	0.29	0.42
τ_00_ cluster	0.38	0.21	0.32	0.57
τ_11_ geographic area	0.04	0.05	0.04	0.04
ρ_01_ geographic area	0.47	0.07	0.47	0.54

1 Measured among respondents who reported having sex in the past 12 months.

2 Measured among respondents who reported knowing that diseases can be transmitted through sexual contact. aOR: adjusted odds ratios; 95% CrI: 95% credible interval; Ref: Reference category; τ_00_: random intercept variance; τ_11_: random slope variance; ρ_01_: correlation between intercept and slope.
