# Supplementary material for: Burden and trends of symptomatic sexually transmitted infections in Malawi from 2000 to 2021: comparative analysis of survey and case report data
Source: Sex Transm Dis. Author manuscript; Available in PMC 2025 May 1. (PMC12044544; doi:10.1097/OLQ.0000000000001919)
Supplement: Supplementary Material [file NIHMS1997396-supplement-Supplementary_Material.docx]

**Burden and trends of symptomatic sexually transmitted infections in Malawi from 2000 to 2021: comparative analysis of survey and case report data**

**Supplementary material**

Julia Michalow^1*^, Andreas Jahn PhD ^2,3,4^, Anne Cori PhD^1^, Prof Marie-Claude Boily^1^, Tiwonge Chimpandule^2,3^, Stone Mbiriyawanda^2^, Washington Ozituosauka^2^, Rose Nyirenda^2^, Jeffrey W Eaton PhD^1,5^

^1^ MRC Centre for Global Infectious Disease Analysis, School of Public Health, Imperial College London, London, United Kingdom

^2^ Malawi Department of HIV, STI & Viral Hepatitis, Ministry of Health, Lilongwe, Malawi

^3^ I-TECH Malawi, Lilongwe, Malawi

^4^ Department of Global Health, University of Washington, Seattle, WA, USA

^5^ Center for Communicable Disease Dynamics, Department of Epidemiology, Harvard T.H. Chan School of Public Health, Boston, MA, USA

* Corresponding author

St. Mary’s Hospital Campus, Norfolk Place, London W2 1PG, United Kingdom j.michalow21@imperial.ac.uk

**Table of Contents**

[Table S1: Variable definitions for self-reported STI, STI symptoms, and STI treatment seeking in Malawi Demographic and Health Surveys in 2000, 2004, 2010, and 2015-16 2](#_Toc149832669)

[Figure S1: Paper STI client register used in health facilities by Department of HIV, STI, and Viral Hepatitis 3](#_Toc149832670)

[Table S2: Socio-demographic characteristics and behaviours of ever sexually active respondents in Demographic and Health Surveys between 2000 and 2015-16 5](#_Toc149832671)

[Table S3: Bayesian generalised linear mixed-effects models on the odds of seeking care or treatment for last infection, among ever sexually active males and females aged 15-54 years who reported an STI or STI symptom in the last 12 months. 6](#_Toc149832672)

[Figure S2: Random effect intercepts on the logit scale for Bayesian models estimating the odds of reporting genital ulcer and genital discharge in the last 12 months among ever sexually active males and females. 7](#_Toc149832673)

[Figure S3: Random effect slopes over time on the logit scale for Bayesian models estimating the odds of reporting genital ulcer and genital discharge in the last 12 months among ever sexually active males and females. 7](#_Toc149832674)

[Figure S4: Random effect intercepts on the logit scale for Bayesian models estimating the odds of seeking care or treatment for last infection among ever sexually active males and females reporting an STI or STI symptom in the last 12 months. 8](#_Toc149832675)

[Figure S5: Random effect slopes over time on the logit scale for models estimating the odds of seeking care or treatment for last infection among ever sexually active males and females reporting an STI or STI symptom in the last 12 months. 8](#_Toc149832676)

[Table S4: Characteristics and diagnoses of STI case reports in the Malawi Department of HIV and AIDS Management Information System during 2014-2021 9](#_Toc149832677)

Table S1: Variable definitions for self-reported STI, STI symptoms, and STI treatment seeking in Malawi Demographic and Health Surveys in 2000, 2004, 2010, and 2015-16

| **Variable** | **Identifier (Questionnaire)** | **Question** |
| --- | --- | --- |
| STI identified in last 12 months | v763a (IR),  mv763a (MR) | Now I would like to ask you some questions about your health in the last 12 months. During the last 12 months, have you had a disease which you got through sexual contact? |
| Genital ulcer in last 12 months (female) | v763b (IR) | Sometimes women have a genital sore or ulcer. During the last 12 months, have you had a genital sore or ulcer? |
| Genital ulcer in last 12 months (male) | mv763b (MR) | Sometimes men have a sore or ulcer near their penis. During the last 12 months, have you had a sore or ulcer on or near your penis? |
| Genital discharge in last 12 months (female) | v763c (IR) | Sometimes women experience a bad-smelling abnormal genital discharge. During the last 12 months, have you had a bad-smelling abnormal genital discharge? |
| Genital discharge in last 12 months (male) | mv763c (MR) | Sometimes men experience an abnormal discharge from their penis. During the last 12 months, have you had an abnormal discharge from your penis? |
| Treatment seeking for STI or STI-related symptom in last 12 months | v770 (IR),  mv770 (MR) | The last time you had (PROBLEM), did you seek any kind of advice or treatment? |
| Sector for treatment of STI or STI-related symptom in last 12 months  *[Only available in DHS 2010 and 2015-16 surveys]* | v770a - x (IR),  mv770a - x (MR) | Where did you go?  Public sector   - Government hospital - Government health centre - Stand along HTC centre - Family planning clinic - Mobile HTC services - Other public sector   Private medical sector   - Private hospital/clinic/private doctor - Stand-alone HTC centre - Pharmacy - Mobile HTC services - Other private medical sector   Other source   - Shop - Other |

IR = Individual women’s recode file, MR: Men’s recode file.


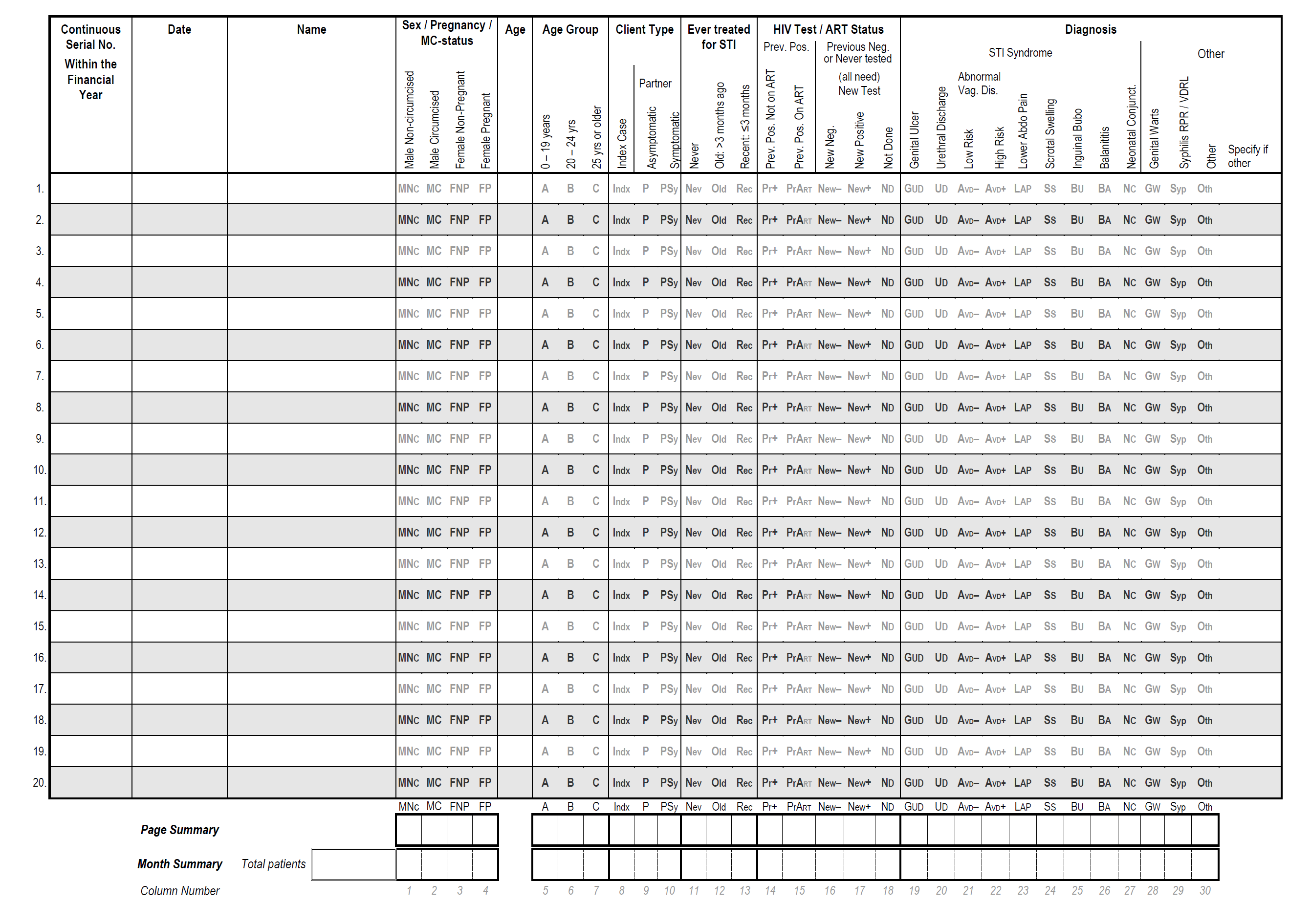


Figure S1: Paper STI client register used in health facilities by Department of HIV, STI, and Viral Hepatitis


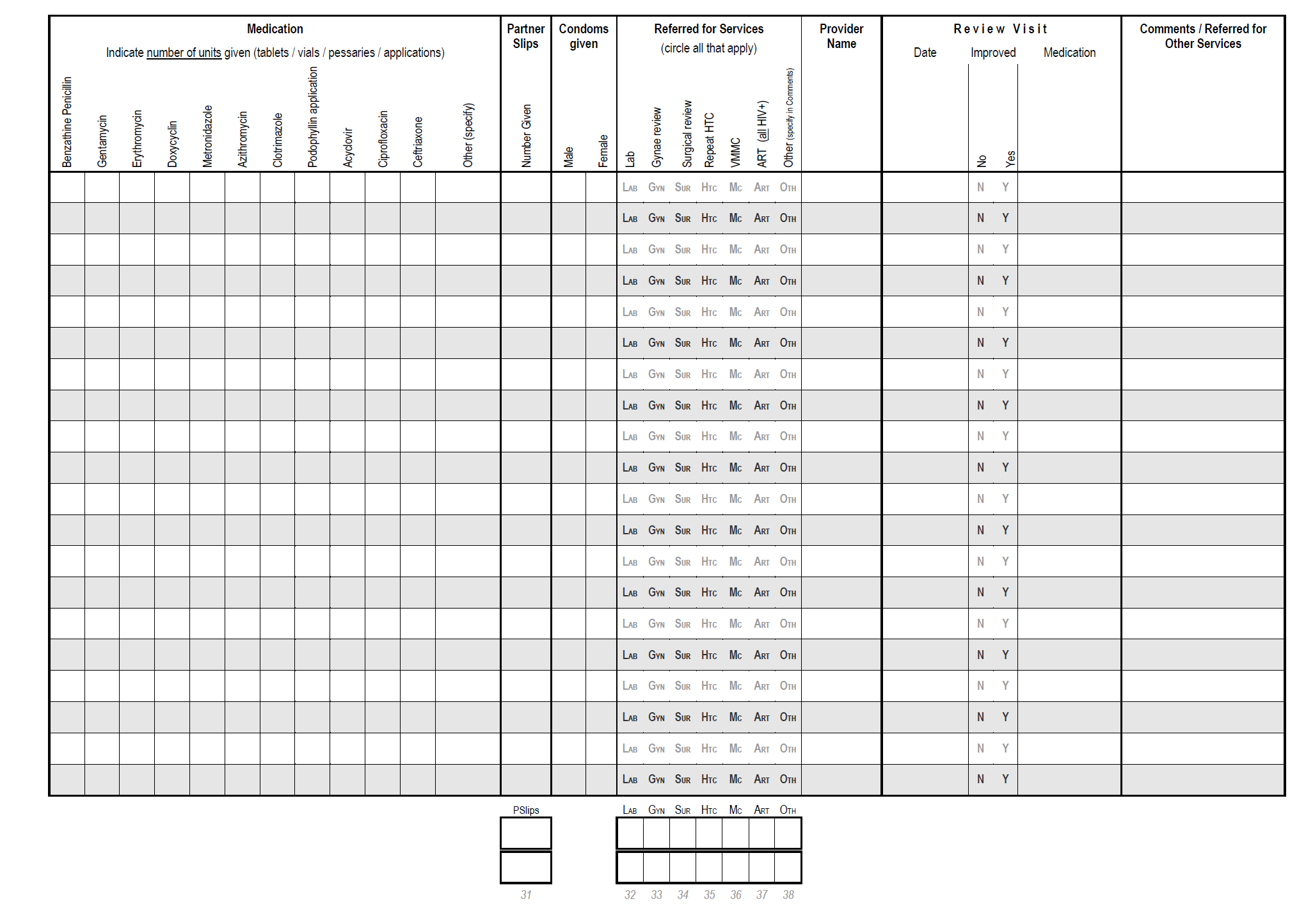


**Figure S1: Paper STI client register used in health facilities by Department of HIV, STI, and Viral Hepatitis**

Table S2: Socio-demographic characteristics and behaviours of ever sexually active respondents in Demographic and Health Surveys between 2000 and 2015-16

|  | **DHS 2000** | | **DHS 2004** | | **DHS 2010** | | **DHS 2015-16** | |
| --- | --- | --- | --- | --- | --- | --- | --- | --- |
|  | **Male**  N = 2 758 | **Female**  N = 11 451 | **Male**  N = 2 816 | **Female**  N = 9 581 | **Male**  N = 5 852 | **Female**  N = 18 201 | **Male**  N = 6 277 | **Female**  N = 20 806 |
| **Age group** |  |  |  |  |  |  |  |  |
| 15-19 | 403 (15%) | 1 676 (15%) | 358 (13%) | 1 277 (13%) | 894 (15%) | 2 109 (12%) | 930 (15%) | 2 630 (13%) |
| 20-24 | 537 (19%) | 2 797 (24%) | 517 (18%) | 2 520 (26%) | 1 007 (17%) | 3 827 (21%) | 1 217 (19%) | 4 639 (22%) |
| 25-29 | 537 (19%) | 2 250 (20%) | 592 (21%) | 1 936 (20%) | 1 008 (17%) | 3 909 (21%) | 997 (16%) | 3 747 (18%) |
| 30-34 | 331 (12%) | 1 504 (13%) | 458 (16%) | 1 331 (14%) | 877 (15%) | 2 966 (16%) | 917 (15%) | 3 488 (17%) |
| 35-39 | 332 (12%) | 1 353 (12%) | 274 (10%) | 996 (10%) | 736 (13%) | 2 314 (13%) | 844 (13%) | 2 870 (14%) |
| 40+ | 618 (22%) | 1 871 (16%) | 617 (22%) | 1 521 (16%) | 1 330 (23%) | 3 076 (17%) | 1 372 (22%) | 3 432 (16%) |
| **Region of residence** |  |  |  |  |  |  |  |  |
| Northern | 460 (17%) | 1896 (17%) | 365 (13%) | 1261 (13%) | 993 (17%) | 3355 (18%) | 1300 (21%) | 4001 (19%) |
| Central | 992 (36%) | 3830 (33%) | 1080 (38%) | 3299 (34%) | 2186 (37%) | 6232 (34%) | 2239 (36%) | 7064 (34%) |
| Southern | 1306 (47%) | 5725 (50%) | 1371 (49%) | 5021 (52%) | 2673 (46%) | 8614 (47%) | 2738 (44%) | 9741 (47%) |
| **Residence type** |  |  |  |  |  |  |  |  |
| Rural | 2 130 (77%) | 9 012 (79%) | 2 397 (85%) | 8 304 (87%) | 5 057 (86%) | 15 864 (87%) | 4 896 (78%) | 16 451 (79%) |
| Urban | 628 (23%) | 2 439 (21%) | 419 (15%) | 1 277 (13%) | 795 (14%) | 2 337 (13%) | 1 381 (22%) | 4 355 (21%) |
| **Marital status** |  |  |  |  |  |  |  |  |
| Never married | 738 (27%) | 905 (7.9%) | 670 (24%) | 653 (6.8%) | 1 611 (28%) | 1 313 (7.2%) | 1 821 (29%) | 2 322 (11%) |
| Formerly married | 124 (4·5%) | 1 500 (13%) | 105 (3·7%) | 1 291 (13%) | 247 (4·2%) | 2 761 (15%) | 258 (4·1%) | 3 139 (15%) |
| Married or living together | 1 896 (69%) | 9 046 (79%) | 2 041 (72%) | 7 637 (80%) | 3 994 (68%) | 14 127 (78%) | 4 198 (67%) | 15 345 (74%) |
| **Highest education** |  |  |  |  |  |  |  |  |
| None | 292 (11%) | 3 166 (28%) | 331 (12%) | 2 425 (25%) | 396 (6·8%) | 2 955 (16%) | 373 (5·9%) | 2 581 (12%) |
| Primary | 1 839 (67%) | 6 986 (61%) | 1 778 (63%) | 5 906 (62%) | 3 697 (63%) | 12 109 (67%) | 3 469 (55%) | 12 776 (61%) |
| Secondary or higher | 627 (23%) | 1 299 (11%) | 707 (25%) | 1 250 (13%) | 1 759 (30%) | 3 137 (17%) | 2 435 (39%) | 5 449 (26%) |
| **Employment** |  |  |  |  |  |  |  |  |
| No or unknown | 972 (35%) | 4 650 (41%) | 1 035 (37%) | 3 893 (41%) | 809 (14%) | 7 287 (40%) | 878 (14%) | 7 120 (34%) |
| Yes | 1 786 (65%) | 6 801 (59%) | 1 781 (63%) | 5 688 (59%) | 5 043 (86%) | 10 914 (60%) | 5 399 (86%) | 13 686 (66%) |
| **Age at first sex** |  |  |  |  |  |  |  |  |
| <15 | 541 (20%) | 2 706 (24%) | 371 (13%) | 2 080 (22%) | 1 108 (19%) | 3 733 (21%) | 1 016 (16%) | 3 908 (19%) |
| 15-19 | 1 530 (55%) | 7 550 (66%) | 1 744 (62%) | 6 545 (68%) | 3 177 (54%) | 12 528 (69%) | 3 675 (59%) | 14 875 (71%) |
| 20-24 | 577 (21%) | 1 088 (9·5%) | 591 (21%) | 884 (9·2%) | 1 296 (22%) | 1 776 (9·8%) | 1 299 (21%) | 1 892 (9·1%) |
| 25+ | 110 (4·0%) | 107 (0·9%) | 110 (3·9%) | 72 (0·8%) | 271 (4·6%) | 164 (0·9%) | 287 (4·6%) | 131 (0·6%) |
| **Number of sex partners in last 12 months**^1^ | | | | | | | | |
| 0 | 291 (11%) | 1 330 (12%) | 299 (11%) | 1 188 (12%) | 799 (14%) | 2609 (14%) | 632 (10%) | 3 080 (15%) |
| 1 | 2 029 (74%) | 10 004 (87%) | 2 211 (79%) | 8 302 (87%) | 4 390 (75%) | 15 472 (85%) | 4 658 (74%) | 17 455 (84%) |
| 2+ | 438 (16%) | 117 (1%) | 306 (11%) | 91 (1%) | 663 (11%) | 120 (1%) | 987 (16%) | 271 (1%) |
| **Condom use at last sex in past 12 months**^1^ | | | | | | | | |
| No or unknown | 2 108 (76%) | 9 609 (84%) | 2 143 (76%) | 7 983 (83%) | 4 025 (69%) | 14 187 (78%) | 4 021 (64%) | 15 641 (75%) |
| Yes | 359 (13%) | 512 (4·5%) | 374 (13%) | 410 (4·3%) | 1 028 (18%) | 1 405 (7·7%) | 1 624 (26%) | 2 085 (10%) |
| Not applicable | 291 (11%) | 1 330 (12%) | 299 (11%) | 1 188 (12%) | 799 (14%) | 2 609 (14%) | 632 (10%) | 3 080 (15%) |
| **Ever tested for HIV** |  |  |  |  |  |  |  |  |
| No or unknown | 2 293 (83%) | 10 364 (91%) | 2 331 (83%) | 8 085 (84%) | 2 487 (42%) | 3 460 (19%) | 1 428 (23%) | 1 807 (9%) |
| Yes | 465 (17%) | 1 087 (9%) | 485 (17%) | 1 496 (16%) | 3 365 (58%) | 14 741 (81%) | 4 849 (77%) | 18 999 (91%) |
| **Knowledge of STIs** |  |  |  |  |  |  |  |  |
| No or unknown | 99 (3·6%) | 970 (8·5%) | 236 (8·4%) | 1 175 (12%) | 315 (5·4%) | 1 310 (7·2%) | 373 (5·9%) | 2 431 (12%) |
| Yes | 2 659 (96%) | 10 481 (92%) | 2 580 (92%) | 8 406 (88%) | 5 537 (95%) | 16 891 (93%) | 5 904 (94%) | 18 375 (88%) |

^1^Measured among respondents who reported having sex in the past 12 months. ^2^Measured among respondents who reported knowing that diseases can be transmitted through sexual contact. Frequencies are unweighted; proportions are survey weighted.

Table S3: Bayesian generalised linear mixed-effects models on the odds of seeking care or treatment for last infection, among ever sexually active males and females aged 15-54 years who reported an STI or STI symptom in the last 12 months.

|  | **Female**  **aOR (95% CrI)** | **Male**  **aOR (95% CrI)** |
| --- | --- | --- |
| **Intercept** | 0.25 (0.16-0.39) | 0.61 (0.19-1.94) |
| **Year** (centred at 2010) | 0.95 (0.93-0.97) | 0.96 (0.92-1.00) |
| **Age group** |  |  |
| 15-19 | 0.64 (0.52-0.79) | 0.47 (0.25-0.86) |
| 20-24 | 0.95 (0.81-1.11) | 0.72 (0.44-1.16) |
| 25-29 | Ref | Ref |
| 30-34 | 1.06 (0.90-1.25) | 1.03 (0.65-1.66) |
| 35-39 | 0.94 (0.79-1.12) | 1.24 (0.76-2.00) |
| 40+ | 1.04 (0.88-1.24) | 1.13 (0.72-1.78) |
| **Residence type** |  |  |
| Rural | Ref | Ref |
| Urban | 1.43 (1.21-1.69) | 1.69 (1.19-2.43) |
| **Region** |  |  |
| Northern | Ref | Ref |
| Central | 1.01 (0.75-1.35) | 0.42 (0.24-0.70) |
| Southern | 1.26 (0.95-1.68) | 0.54 (0.31-0.88) |
| **Marital status** |  |  |
| Never married | Ref | Ref |
| Formerly married | 1.36 (1.04-1.79) | 1.87 (0.96-3.70) |
| Married or living together | 1.19 (0.92-1.53) | 1.28 (0.79-2.17) |
| **Highest education** |  |  |
| None | Ref | Ref |
| Primary | 1.16 (1.01-1.34) | 1.24 (0.72-2.18) |
| Secondary or higher | 1.37 (1.13-1.68) | 1.13 (0.62-2.11) |
| **Employment** |  |  |
| No or unknown | Ref | Ref |
| Yes | 1.01 (0.90-1.13) | 1.05 (0.72-1.57) |
| **Age at first sex** |  |  |
| <15 | 1.09 (0.96-1.23) | 1.27 (0.89-1.78) |
| 15-19 | Ref | Ref |
| 20-24 | 0.87 (0.71-1.05) | 1.2 (0.81-1.80) |
| 25+ | 0.95 (0.49-1.86) | 1.65 (0.73-3.82) |
| **Number of sex partners in past year**^1^ |  |  |
| None | Ref | Ref |
| 1 | 1.07 (0.88-1.30) | 1.42 (0.85-2.37) |
| 2+ | 1.28 (0.86-1.89) | 2.16 (1.23-3.76) |
| **Condom use at last sex in past year**^1^ |  |  |
| No or unknown | Ref | Ref |
| Yes | 1.23 (1.03-1.50) | 0.9 (0.62-1.32) |
| **Ever tested for HIV** |  |  |
| No or unknown | Ref | Ref |
| Yes | 1.48 (1.26-1.73) | 1.53 (1.12-2.08) |
| **Knowledge of STIs** |  |  |
| No or unknown | Ref | Ref |
| Yes | 1.5 (1.25-1.79) | 0.82 (0.42-1.62) |
| **Symptom** |  |  |
| None | 1.18 (0.94-1.47) | 1.19 (0.70-2.04) |
| Discharge | Ref | Ref |
| Ulcer | 1.7 (1.50-1.94) | 1.14 (0.82-1.59) |
| Discharge and ulcer | 2.39 (2.04-2.80) | 2.65 (1.74-4.06) |
| **Random effects** |  |  |
| τ_00_ geographic area | 0.23 | 0.11 |
| τ_00_ cluster | 0.21 | 0.82 |
| τ_11_ geographic area | 0.03 | 0.07 |
| ρ_01_ geographic area | 0.22 | 0.16 |

^1^Measured among respondents who reported having sex in the past 12 months. ^2^Measured among respondents who reported knowing that diseases can be transmitted through sexual contact. aOR: adjusted odds ratios; 95% CrI: 95% credible interval; Ref: Reference category; τ_00_: random intercept variance; τ_11_: random slope variance; ρ_01_: correlation between intercept and slope.


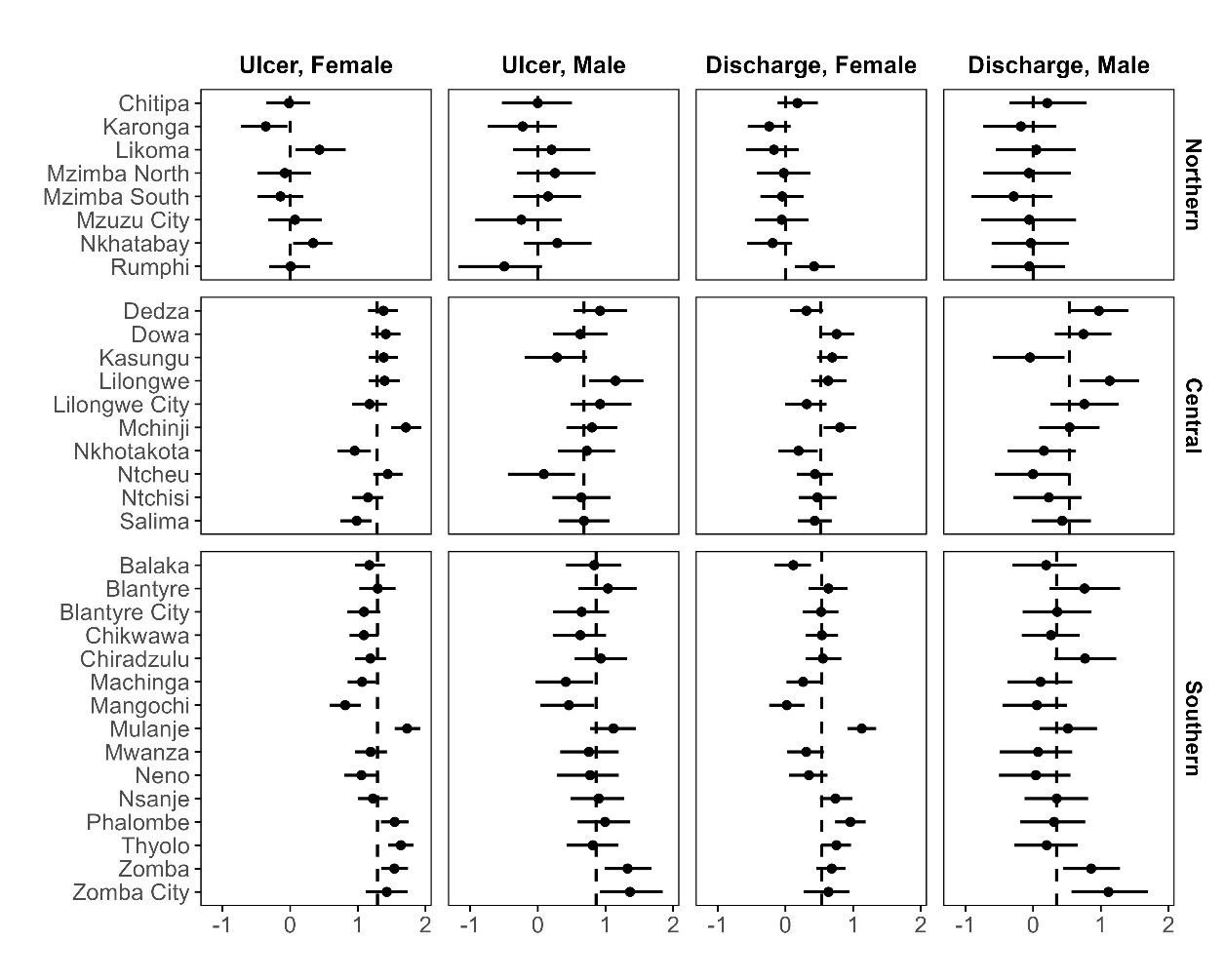


Figure S2: Random effect intercepts on the logit scale for Bayesian models estimating the odds of reporting genital ulcer and genital discharge in the last 12 months among ever sexually active males and females.

Random intercepts are offset by regional fixed effects. Error bars represent 95% credible intervals.


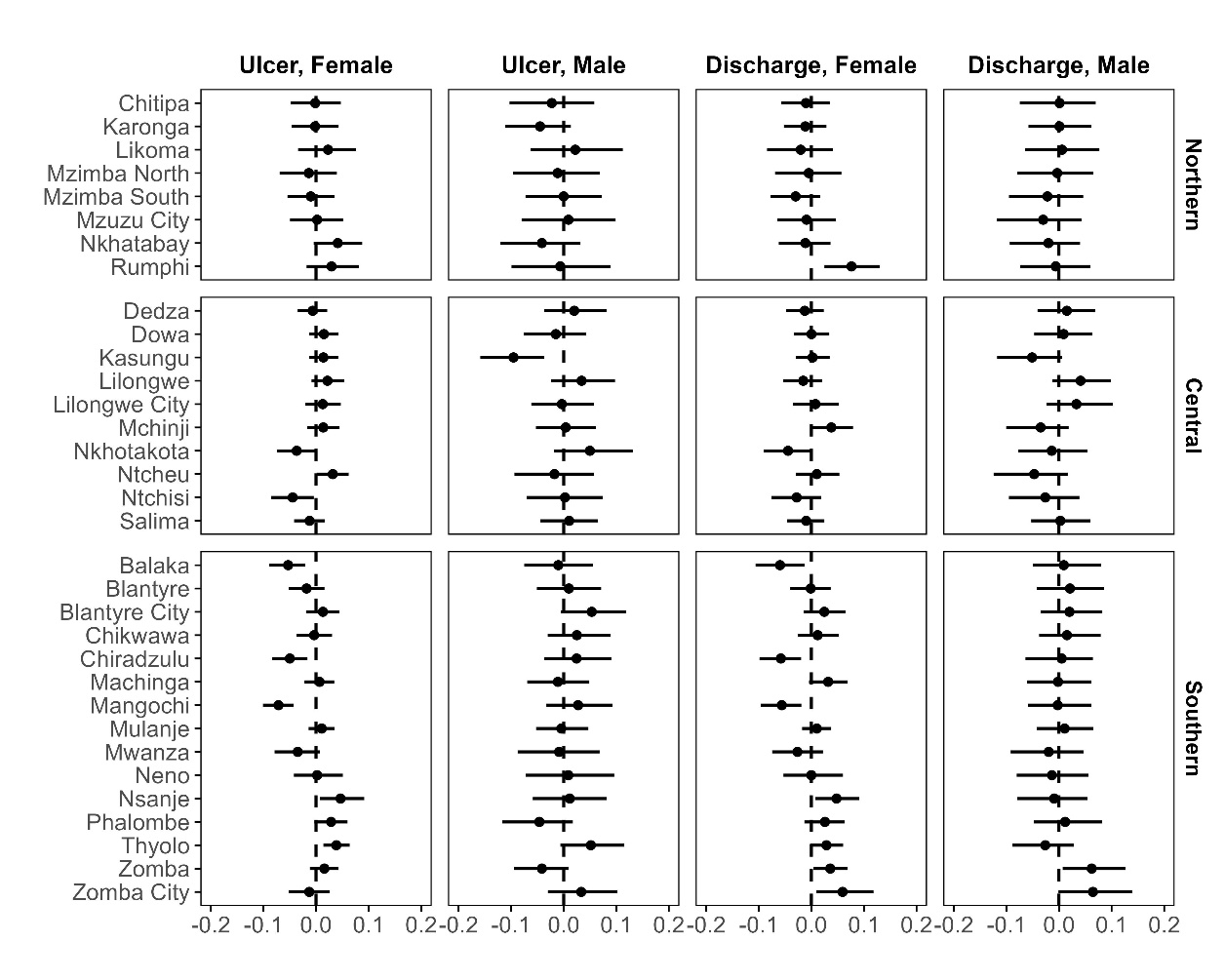


Figure S3: Random effect slopes over time on the logit scale for Bayesian models estimating the odds of reporting genital ulcer and genital discharge in the last 12 months among ever sexually active males and females.

Error bars represent 95% credible intervals.


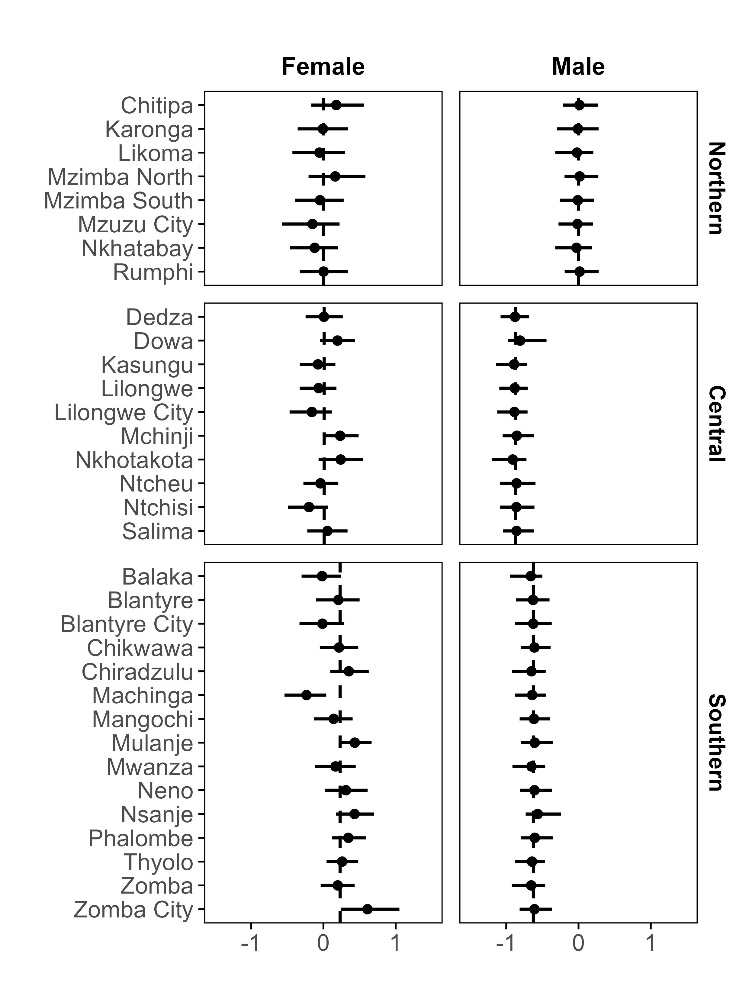


Figure S4: Random effect intercepts on the logit scale for Bayesian models estimating the odds of seeking care or treatment for last infection among ever sexually active males and females reporting an STI or STI symptom in the last 12 months.

Random intercepts are offset by regional fixed effects. Error bars represent 95% credible intervals.


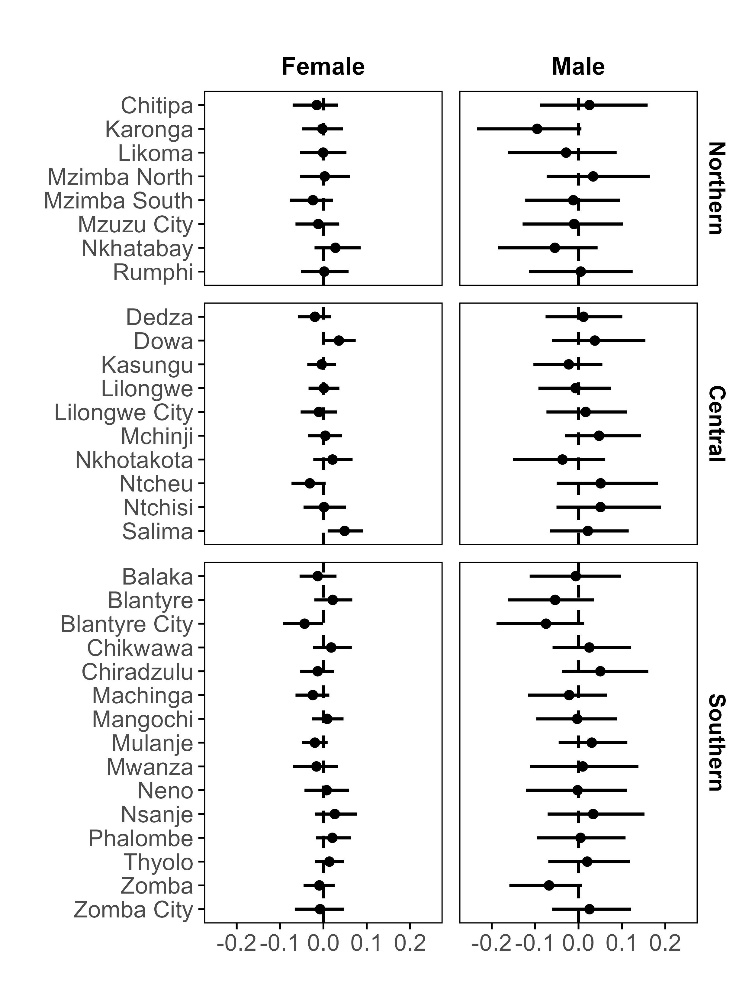


Figure S5: Random effect slopes over time on the logit scale for models estimating the odds of seeking care or treatment for last infection among ever sexually active males and females reporting an STI or STI symptom in the last 12 months.

Error bars represent 95% credible intervals.

Table S4: Characteristics and diagnoses of STI case reports in the Malawi Department of HIV and AIDS Management Information System during 2014-2021

|  | **2014**  N = 212 404 | **2015**  N = 227 144 | **2016**  N = 264 493 | **2017**  N = 304 341 | **2018**  N = 355 236 | **2019**  N = 413 142 | **2020**  N = 386 346 | **2021**  N = 384 631 | **Total**  N = 2 547 737 |
| --- | --- | --- | --- | --- | --- | --- | --- | --- | --- |
| **Sex** |  |  |  |  |  |  |  |  |  |
| Male | 85 670 (40%) | 91 239 (40%) | 107 431 (41%) | 124 046 (41%) | 142 020 (40%) | 164 432 (40%) | 157 834 (41%) | 159 721 (42%) | 1 032 393 (41%) |
| Female | 126 734 (60%) | 135 905 (60%) | 157 062 (59%) | 180 295 (59%) | 213 216 (60%) | 248 710 (60%) | 228 512 (59%) | 224 910 (58%) | 1 515 344 (59%) |
| **Age group** |  |  |  |  |  |  |  |  |  |
| 0 - 19 | 18 696 (8·7%) | 20 314 (8·9%) | 23 960 (9·1%) | 27 857 (9·2%) | 31 091 (8·8%) | 37 104 (9·0%) | 31 341 (8·1%) | 32 616 (8·5%) | 222 979 (8·8%) |
| 20 - 24 | 50 908 (24%) | 54 436 (24%) | 63 756 (24%) | 72 578 (24%) | 84 780 (24%) | 92 849 (22%) | 89 163 (23%) | 91 298 (24%) | 599 768 (24%) |
| 25+ | 142 800 (67%) | 152 394 (67%) | 176 777 (67%) | 203 906 (67%) | 239 365 (67%) | 283 189 (69%) | 265 842 (69%) | 260 717 (68%) | 1 724 990 (68%) |
| **Region** |  |  |  |  |  |  |  |  |  |
| Central | 71 067 (33%) | 78 300 (34%) | 88 426 (33%) | 105 197 (35%) | 122 507 (34%) | 143 486 (35%) | 136 333 (35%) | 141 688 (37%) | 887 004 (35%) |
| Northern | 27 614 (13%) | 31 395 (14%) | 35 424 (13%) | 40 793 (13%) | 48 862 (14%) | 54 558 (13%) | 48 661 (13%) | 51 462 (13%) | 338 769 (13%) |
| Southern | 113 723 (54%) | 117 449 (52%) | 140 643 (53%) | 158 351 (52%) | 183 867 (52%) | 215 098 (52%) | 201 352 (52%) | 191 481 (50%) | 1 321 964 (52%) |
| **HIV status** |  |  |  |  |  |  |  |  |  |
| HIV-positive | 27 098 (13%) | 31 327 (14%) | 42 568 (16%) | 48 406 (16%) | 56 638 (16%) | 67 204 (16%) | 64 617 (17%) | 62 844 (16%) | 400 702 (16%) |
| HIV-negative | 77 484 (36%) | 93 963 (41%) | 152 706 (58%) | 205 193 (67%) | 257 466 (72%) | 301 261 (73%) | 283 883 (73%) | 280 812 (73%) | 1 652 768 (65%) |
| Unknown | 107 822 (51%) | 101 854 (45%) | 69 219 (26%) | 50 742 (17%) | 41 132 (12%) | 44 677 (11%) | 37 846 (10%) | 40 975 (11%) | 494 267 (19%) |
| **Client type** |  |  |  |  |  |  |  |  |  |
| Symptomatic index | 169 686 (80%) | 183 836 (81%) | 214 733 (81%) | 249 298 (82%) | 291 509 (82%) | 336 625 (81%) | 316 069 (82%) | 316 910 (82%) | 2 078 666 (82%) |
| Symptomatic partner | 17 253 (8·1%) | 17 788 (7·8%) | 21 319 (8·1%) | 22 036 (7·2%) | 23 414 (6·6%) | 27 343 (6·6%) | 27 626 (7·2%) | 27 556 (7·2%) | 184 335 (7%) |
| Asymptomatic partner | 25 465 (12%) | 25 520 (11%) | 28 441 (11%) | 33 007 (11%) | 40 313 (11%) | 49 174 (12%) | 42 651 (11%) | 40 165 (10%) | 284 736 (11%) |
| **STI treatment history** |  |  |  |  |  |  |  |  |  |
| ≤ 3 months | 18 232 (8·6%) | 17 996 (7·9%) | 18 763 (7·1%) | 20 964 (6·9%) | 23 197 (6·5%) | 28 165 (6·8%) | 25 287 (6·5%) | 23 956 (6·2%) | 176 560 (7·2%) |
| > 3 months | 34 589 (16%) | 40 735 (18%) | 47 856 (18%) | 52 887 (17%) | 65 988 (19%) | 77 254 (19%) | 74 946 (19%) | 76 558 (20%) | 470 813 (18%) |
| Never treated | 159 583 (75%) | 168 413 (74%) | 197 874 (75%) | 230 490 (76%) | 266 051 (75%) | 307 723 (74%) | 286 113 (74%) | 284 117 (74%) | 1 900 364 (75%) |
| **Diagnosis^1^** |  |  |  |  |  |  |  |  |  |
| UDS | 52 276 (23%) | 55 586 (23%) | 68 718 (25%) | 79 165 (25%) | 91 163 (25%) | 106 724 (25%) | 109 412 (27%) | 112 790 (28%) | 675 834 (25%) |
| VDS high risk | 41 117 (18%) | 44 350 (19%) | 51 371 (19%) | 62 160 (20%) | 79 740 (22%) | 92 707 (22%) | 87 657 (22%) | 86 834 (22%) | 545 936 (21%) |
| VDS low risk | 25 090 (11%) | 28 496 (12%) | 30 802 (11%) | 32 325 (10%) | 35 375 (9·6%) | 37 633 (8·8%) | 32 655 (8·2%) | 32 903 (8·2%) | 255 279 (10%) |
| GUD | 37 158 (17%) | 40 625 (17%) | 44 073 (16%) | 47 748 (15%) | 50 686 (14%) | 55 480 (13%) | 48 309 (12%) | 48 877 (12%) | 372 956 (14%) |
| LAP | 36 316 (16%) | 36 796 (15%) | 41 164 (15%) | 47 077 (15%) | 54 571 (15%) | 56 732 (13%) | 49 842 (12%) | 46 986 (12%) | 369 484 (14%) |
| Other^2^ | 30 943 (14%) | 33 930 (14%) | 40 075 (15%) | 48 415 (15%) | 57 656 (16%) | 80 863 (19%) | 71 842 (18%) | 73 861 (18%) | 437 585 (16%) |
| Total | 222 900 (100%) | 239 784 (100%) | 276 203 (100%) | 316 890 (100%) | 369 191 (100%) | 430 139 (100%) | 399 718 (100%) | 402 251 (100%) | 2 657 076 (100%) |
| **Health sector** |  |  |  |  |  |  |  |  |  |
| Public | 207 872 (98%) | 218 911 (96%) | 254 844 (96%) | 292 670 (96%) | 342 965 (97%) | 40 0826 (97%) | 375 488 (97%) | 371 050 (96%) | 2 464 626 (97%) |
| Private | 4 532 (2.1%) | 8 233 (3.6%) | 9 649 (3.6%) | 11 671 (3.8%) | 12 271 (3.5%) | 12 316 (3%) | 10 858 (2.8%) | 13 581 (3.5%) | 83 111 (3·3%) |

^1^Multiple diagnoses possible per STI case. ^2^Other diagnosis includes balanitis, genital warts, inguinal bubo, neonatal conjunctivitis, scrotal swelling, syphilis, among others. UDS: urethral discharge syndrome; VDS: vaginal discharge syndrome; GUD: genital ulcer disease; LAP: lower abdominal pain.
